# Mapping the roots of specialist disparities—Authors’ reply

**DOI:** 10.1016/j.lanepe.2023.100815

**Published:** 2023-12-15

**Authors:** Lianne Mulder, Anouk Wouters, Eddymurphy U. Akwiwu, Andries S. Koster, Saskia M. Peerdeman, Mahdi Salih, Rashmi A. Kusurkar

**Affiliations:** a Amsterdam UMC Location Vrije Universiteit Amsterdam, Research in Education, De Boelelaan 1118, Amsterdam, the Netherlands; b LEARN! Research Institute for Learning and Education, Faculty of Psychology and Education, VU University, Amsterdam, the Netherlands; c Amsterdam UMC, Vrije Universiteit Amsterdam, Epidemiology and Data Science, Amsterdam Public Health, De Boelelaan 1117, Amsterdam, the Netherlands; d Department of Pharmaceutical Sciences, Utrecht University, David de Wied Building, Universiteitsweg 99, Utrecht, the Netherlands; e Amsterdam UMC Location University of Amsterdam, Department of Neurosurgery, Meibergdreef 9, Amsterdam, the Netherlands; f Amsterdam UMC Location University of Amsterdam, Faculty of Medicine, Teaching and Learning Centre, Meibergdreef 9, Amsterdam, the Netherlands; g Amsterdam Public Health, Quality of Care, Amsterdam, the Netherlands; h Erasmus MC, Division of Nephrology and Transplantation, Department of Internal Medicine, Dr. Molewaterplein 40, Rotterdam, The Netherlands

We appreciate the interest of Hemant Sharma in our recent study on the loss of diversity in the pathway from medical student to specialist in The Netherlands,^1^ and we welcome the opportunity to address the points about our methodology and the theory used.

First, student populations have indeed changed since 2002–2004, but students in this cohort have become the specialists of today, so the analysis is very much contemporary. The choice of this cohort was related to accommodating the time required to become a specialist in the fields with the lengthiest residency programs. That said, we fully acknowledge the limitation of our study that it does not represent the current medical student population. Demographic data of the first-year medical student population in 2019 and 2020 can be found in Mulder et al.^2^ We aim to replicate our study several times in the coming years, each time with a new cohort of first-year medical students. This would enable us to do a trend analysis, and to see whether changes in the student population, with different views on working as a medical professional and work-life balance, are reflected in the physician and specialist populations.

Second, we indeed did not examine the selection process, because there is no national application system for residency training positions. Therefore, we have recommended the implementation of such a system, as (inter)national research suggests that both self-selection and inequality of opportunity exist in the recruitment and selection for residency training programs. We look forward to studying both elements with national data once the data are available. This would enable us to study whether cultural cloning patterns can be witnessed in application rates and acceptance rates, in line with Nguemeni Tiako et al.^3^ and our own study.

Third, the suggested variables (race, disability, sexual orientation) are not registered by Statistics Netherlands, therefore they could not be included in the analyses. On a more critical note, it is well-established that ‘race’ is a social construct, and not a biological entity,^4^ so ‘race’ would anyway not be a part of our analysis. The belief in different human ‘races’ as genetically real is the foundation for the existence and perpetuation of racism. However, we do agree that additional variables beyond the six that we included in our paper, would enhance the context-based intersectional understanding of cultural cloning. For example, in various contexts, influential variables in career progression may be public or private high school attendance, membership in elite or honor associations, religion, caste, refugee status, or Indigenous status.

Fourth, we agree that it is difficult to measure cultural cloning quantitatively. Our approach is the first attempt to do so in the Dutch medical context. We encourage other researchers to engage in follow-up studies, both quantitatively and qualitatively, to deepen our collective understanding of the processes underlying the differences in career progression between different groups of people. For example, our data showed the largest differences in specialization rates on a national level between male physicians without a migration background (74.4%), and female physicians with a Turkish or Moroccan migration background (42.7%). To investigate how a lack of mentors and stereotype threat (as suggested by Hemant Sharma) may contribute to cultural cloning, qualitative methods would be appropriate. For example, social network analysis may be well-suited to study (differential) access to mentors. Biased grading has been observed in the Dutch context by Van Andel et al.^5^

Fifth, we would like to clarify that it is not our country’s data itself that we generalized to cultural cloning processes globally. We agree that racism and exclusion manifest differently across contexts. Therefore, we did not attempt to fit other settings into the theoretical model of cultural cloning, but studied whether cultural cloning can be witnessed in the nationwide data from the Netherlands. It is only our methodology that we argued can be applied in other (international) contexts, to test whether cultural cloning can be seen in the data resulting from the performed analyses, using variables that are relevant in each context.

## Contributors

LM wrote the first draft. AW, EUA, SP, MS, ASK and RAK contributed to reviewing and editing the response letter. LM wrote the final draft.

## Declaration of interests

No conflict of interests.
